# Whole-body MRI and diffusion MRI

**DOI:** 10.1186/1470-7330-14-S1-O31

**Published:** 2014-10-09

**Authors:** Anwar R Padhani

**Affiliations:** 1 Mount Vernon Cancer Centre, London, UK

## 

Metastatic bone disease is a common manifestation of advanced cancers with a very high prevalence in breast, prostate and lung cancers. In order to effectively manage patients with metastatic bony disease it is essential to have consistent, reproducible and validated methods for detecting and assessing the response to therapy. In response assessments, confident categorization of response is needed to enable therapeutic strategies to be tailored to individual patient’s benefit. Commonly used methods such as x-rays, bone and CT scans do not always enable the positive assessment of therapeutic benefit to be made but instead provide an evaluation of progression, which then guides therapy decisions in the clinic.

Whole body MRI incorporating DW imaging (WB-DWI) has emerged as a promising bone marrow assessment tool [1] for detection [2,3] and therapy monitoring of bone metastases [4]. On WB-DWI, lytic/infiltrative skeletal metastases appear as focal or diffuse areas of high-signal intensity on high b-values on a background of lower signal intensity of the normal bone marrow [4,5]. Metastasis detection with DWI should be done with anatomical MRI [1,4,5]; a recent meta-analysis demonstrated high sensitivity of WB-DWI to detect metastases at the expense of specificity [2]. Causes for false-positive findings on WB-DWI include bone marrow oedema, vertebral haemangioma, isolated bone marrow islands and bone marrow hyperplasia. False-negative findings occur when there are low levels of bone marrow infiltration or when background bone marrow hyperplasia obscures metastases. Detection of skeletal metastases may be impaired in areas of body movement and the visibility of skull base infiltrations is impaired because of the adjacent high signal of the brain. False-negative findings also include treated malignant disease and sclerotic deposits.

WB-DWI when combined with emerging “wet” biomarkers can improve the classification of therapy response in patients with metastatic bone disease. Both high b-value image signal intensity and ADC value changes are needed for therapeutic assessments [4,5]. A range of imaging findings can be seen depending on the type of therapy and duration of treatment [4]. Morphologic and diffusion MRI therapy response criteria need to be tested in prospective studies in order to address current, unmet clinical and pharmaceutical needs for reliable measures of tumor response in metastatic bone disease [5].
